# A Long‐Term Projection for Emergency Ambulance Services Demand in Taipei and the Related Effects by Temperature

**DOI:** 10.1155/emmi/1072443

**Published:** 2026-01-19

**Authors:** Ho Ting Wong, Tuan-Duong Nguyen

**Affiliations:** ^1^ Department of Business Administration, National Taiwan Normal University, Taipei, Taiwan, ntnu.edu.tw; ^2^ Department of Taiwanese Literature, National Cheng Kung University, Tainan, Taiwan, ncku.edu.tw; ^3^ Institute of Graduate Studies and Leadership, Hoa Sen University, Ho Chi Minh City, Vietnam

**Keywords:** aging population, emergency ambulance services, long-term projection, Taiwan, temperature, weather

## Abstract

**Research Purpose:**

This study aims to project the yearly emergency ambulance service (EAS) demand for Taipei from 2015 to 2051. The effects of daily average temperature on EAS demand in 2036 and 2051 will also be projected.

**Research Methods:**

Over 140,000 EAS patient records in 2015 were obtained from the Taipei Fire Department in order to conduct the EAS demand projection. The projection was computed accounting for changes in the age‐gender structure compared to the base year (2015). The relationship between daily average temperature and EAS demand in 2036 and 2051 was further explored by including and excluding age‐gender structure changes.

**Results:**

Without accounting for changes in the age‐gender structure, the 2051 EAS demand for age groups over 65 was consistently underestimated by 42%–90%, while that for younger age groups was overestimated by 35%–55%. In addition, the projected quadratic curve for describing the relationship between average daily temperature and EAS demand in 2051 showed a significant upward shift and increase in curvature when accounting for changes in the age‐gender structure.

**Conclusions:**

With an accurate long‐term projection for EAS demand in Taipei city and other regions in Taiwan, the government can design strategies for improving the EAS system in order to deal with the rapidly aging population.

## 1. Introduction

### 1.1. Global Population Aging and Healthcare Challenges

Population aging is accelerating across the globe, fundamentally transforming demographic structures and posing significant challenges for healthcare systems. According to the World Population Prospects 2019, one in six people in the world will be aged over 65 by 2050, compared to one in 11 in 2019. In terms of population, there were more than 703 million people aged 65 years or over worldwide in 2019. By 2050, this number is projected to double, reaching approximately 1.5 billion [1]. The phenomenon of population aging is particularly pronounced in Latin America, the Caribbean, and Eastern and South‐Eastern Asia. For example, the proportion of elderly people aged 65 or above within the total population in Latin America and the Caribbean increased from 5% in 1990 to 9% in 2010, and Eastern and South‐Eastern Asia exhibited a similar trend, rising from 6% in 1990 to 11% in 2019 [1].

These demographic shifts are linked to various challenges for public services and the broader social climate. A swelling elderly population is associated with labor shortages and increased costs for social welfare, significantly challenging healthcare systems irrespective of a country’s income level [2–7]. In response, governments around the world have implemented innovative solutions such as pension system reforms, the development of age‐friendly cities, revised healthcare financing strategies, and the introduction of digital health innovations [5, 8, 9]. In populous Asian countries with rapidly aging populations, including Japan [10], Korea [3], China [11], India [11], and Taiwan [5], the impact of the expanding older population on healthcare systems is especially intense. Elderly people are associated with many physical and emotional problems; they tend to suffer more and more illnesses—including chronic and life‐threatening diseases [12–14]—will contribute substantially to future healthcare demand and expenditure [7, 15]. According to the Centers for Medicare and Medicaid Services, the United States spent over 15% of its GDP on national health expenditure in 2016, and this figure is estimated to rise to 19.4% (approximately $6 trillion) by 2027 [16]. With healthcare demand increasingly outpacing supply, projecting the future needs of healthcare systems becomes ever more complex [17]. Both academic leaders and policy makers must scrutinize and adapt their healthcare systems to ensure sustainability and holistic coverage on national and regional scales, especially as populations age and undergo demographic transitions.

### 1.2. Emergency Ambulance Services (EASs) in an Aging Society

Among the specific domains of health services impacted by population aging, EASs are of critical concern [18, 19]. The growing demand for EAS is recognized as one of the most significant consequences of population aging. Planning and managing resources to address EAS demand—particularly among elderly populations—will become even more critical in coming decades. Changing weather patterns and global warming further complicate the challenge, as both short‐term[20] and long‐term [18] EAS demands are shaped not only by demographic changes but also by environmental conditions. Extreme weather events, which are becoming more difficult to predict due to climate change, have notable negative impacts on human health and community safety. Elderly people, along with children, low‐income populations, and women, are especially vulnerable to such events [20].

### 1.3. Forecasting Emergency Service Demand: Research Progress and Gaps

There is a growing body of research on EAS demand forecasting in various countries, including Australia [21], Canada [22], United Kingdom [23], and Hong Kong [19]. Some of these studies have incorporated weather variables to improve short‐term forecasting accuracy, as seen in Hong Kong and Australia [19, 21]. However, most previous research has focused on EAS demand projections for short‐term periods, typically within a single year, and few studies have investigated long‐term projections spanning a decade or more, particularly those that integrate relevant demographic and environmental factors [7, 24, 25]. A long‐term projection model that combines these elements for a specific region would offer substantial benefits for preparing and planning responses to both population aging and climate change.

### 1.4. The Case of Taiwan and Taipei

Taiwan exemplifies these demographic dynamics, with more than 3.6 million people aged 65 years or older in 2019, accounting for 15.1% of the population. These numbers are projected to reach 5.6 million and 23.4% by 2030 [1]. Notably, Taiwan is among the countries with the fastest rates of population aging, averaging a 0.6% annual increase since 2006. Taiwan is expected to become a super‐aged society by 2026, with 20% or more of the population aged 65 or older [1, 26].

Taipei city, as an international metropolis with a population of 2.7 million in 2019, faces particularly acute challenges. In 2015, 14.7% of Taipei’s population was aged 65 years or older. By 2020, this proportion exceeded 18% and is projected to surpass 27% by 2030, far above the national average. There is also a notable gender imbalance among Taipei’s elderly population, with women outnumbering men in older age groups, particularly among those aged 85 and above [5]. In recognition of these challenges, the Taipei government has implemented strategies aimed at enhancing elderly care, including improvements in general medical services, the development of geriatrics training programs, the expansion of post‐acute services, and the strengthening of linkages between health and social care systems [5].

Despite these policy efforts, the sustainability of EAS in Taipei faces new threats from rapid demographic shifts. For example, the number of ambulance calls in Taipei rose from approximately 97,000 in 2007 to 141,000 in 2016—an increase of nearly 50% over a decade. Over the same period, the EAS workforce grew from 1,761 to 2,063 personnel, a rise of only 17% [27]. This disparity reveals a growing imbalance between EAS demand and workforce capacity. Consequently, the Taipei City Fire Department, which oversees EAS, must identify cost‐effective solutions to increase its management capability and keep pace with rising demand.

### 1.5. Study Objectives

Given these circumstances, there is a clear and urgent need for advanced and integrated approaches to long‐term EAS demand projection. Most previous research has focused on short‐term forecasts or has failed to incorporate key demographic and weather‐related factors. Developing and applying models that consider both population structure and climate variability will better equip policymakers to respond to the challenges posed by an aging society and environmental change.

The main purpose of this article is to present a long‐term projection for yearly EAS demand from 2015 to 2051 in Taipei city, taking into consideration the age‐gender structure change using the method developed by Lai and Wong [24]. Furthermore, the effect of temperature on daily EAS demand in Taipei during this period will also be projected using the same method. This approach was selected because the dataset available for this study aligns well with the methodological requirements. Additionally, comprehensive documentation of the algorithm and parameters facilitates a direct comparison of results between Taipei and Hong Kong. The findings of this study aim to support the development of effective strategies for improving the EAS system, enabling Taipei and similar regions to address the dual challenges of population aging and climate change.

## 2. Methods

### 2.1. Data

Three types of data were used in this research: EAS patient records; daily average temperature time series data; and age‐gender–specific population projection data. First, over 140,000 EAS patient records were obtained from the Taipei City Fire Department of the Taipei City Government through e‐mail request [28]. An onsite presentation of the project was given to the Taipei Fire Department and the request was finally approved. The time period of the records covers the whole of 2015, and each record includes information on patients’ age and gender. Before conducting the projection, the records were aggregated into age‐gender–specific daily EAS demand statistics. The year 2015 was selected as the reference year because it represents the most recent period with reliable and stable data availability. Data from 2019 onwards were potentially influenced by the COVID‐19 pandemic and thus should be excluded to avoid bias. To verify that 2015 was not an outlier, we retrieved total EAS demand data from the four preceding years (2011–2014) and conducted Grubbs’ test for outlier detection. The results showed no statistically significant outliers (*p* > 0.05), indicating that 2015 is a representative year (Table 1). Detailed profile of the yearly EAS demand statistic from 2011–2015 is shown as Table 2.

**Table 1:** Result of Grubbs’ test on yearly EAS demand.

Year	Total EAS demand	*Z*‐statistics	Significant outlier
2011	1,41,250	0.01	No
2012	1,40,153	0.37	No
2013	1,36,988	1.45	Furthest from the rest, but not a significant outlier (*p* > 0.05)
2014	1,43,243	0.69	No
2015	1,44,539	1.13	No

**Table 2:** Detailed profile of the yearly EAS demand statistics from 2011–2015.

Category/year	2011	2012	2013	2014	2015
Age below 15	2243 (2.0)	2275 (1.9)	2490 (2.0)	2636 (2.1)	2521 (2.0)
Age 15–34	28,465 (25.5)	29,990 (25.2)	31,760 (25.7)	33,135 (25.8)	32,513 (25.4)
Age 35–64	41,930 (37.5)	45,520 (38.3)	47,804 (38.6)	48,085 (37.5)	47,328 (37.0)
Age 65+	39,081 (35.0)	40,995 (34.5)	41,681 (33.7)	44,513 (34.7)	45,442 (35.6)

Triage level 1	8369 (8.7)	8845 (9.1)	8440 (8.7)	8984 (9.0)	8786 (8.9)
Triage level 2	18,895 (19.6)	19,182 (19.7)	18,052 (18.6)	19,226 (19.2)	20,914 (21.1)
Triage level 3	51,949 (53.8)	56,190 (57.8)	57,573 (59.3)	60,036 (59.9)	59,600 (60.1)
Triage level 4	16,360 (16.9)	12,080 (12.4)	12,136 (12.5)	11,189 (11.2)	9317 (9.4)
Triage level 5	1025 (1.1)	989 (1.0)	916 (0.9)	757 (0.8)	609 (0.6)

Female	46,272 (41.3)	48,590 (40.8)	50,394 (40.7)	53,274 (41.5)	53,383 (41.8)
Male	65,880 (58.7)	70,437 (59.2)	73,345 (59.3)	75,124 (58.5)	74,424 (58.2)

Trauma	61,312 (43.4)	61,068 (43.6)	60,919 (44.5)	64,914 (45.3)	64,892 (44.9)
Nontrauma	79,938 (56.6)	79,085 (56.4)	76,069 (55.5)	78,329 (54.7)	79,647 (55.1)

*Note:* The sum of each category may not be the same due to missing data; the statistics presented were computed using data from ambulance usage records in this study.

Second, daily average temperature data were obtained from the Central Weather Bureau through the “Climate Observation Data Inquire Service” system [29]. Among the 16 official weather stations in Taipei city, the Taipei weather station was selected due to data completeness, as well as the station’s location; it is close to sea level and within the Taipei metropolitan area. The data are in a time series format that covers the same period as the EAS patient records. Finally, the age‐gender–specific population projection was obtained from the final report of the official population projection provided by the Taipei City Government [30]. In contrast to the EAS patient records and average temperature data, the projection period covers 34 years from 2015 to 2051.

### 2.2. Data Analysis

#### 2.2.1. Projection Method of Yearly EAS Demand From 2015 to 2051

Each official projection of the population pyramids from 2015 to 2051 was first divided, elementwise, by the population pyramid of the base year (i.e., 2015). Following this division, the age‐gender–specific population change indices for Taipei’s population structure within the study period were obtained. By further calculating the cross‐product of 2015’s age‐gender–specific EAS demand and age‐gender–specific population change index vectors, the expected growth of the EAS demand within the study period (i.e., 2015–2051), taking into consideration the age‐gender structure change, could then be obtained. In short, the projection can be summarized as a weighted linear combination of the age‐gender–specific EAS demands, where the weights are the age‐gender–specific population ratio between the projection target year and the base year. Mathematically it can be written as follows:

(1) Yprojection target year=∑age ∑genderWage group,genderYi,gender,base year.

#### 2.2.2. Projection Method of Weather Effects on Daily EAS Demand From 2015 to 2051

First, multivariate quadratic regression was used to derive a set of age‐gender–specific temperature‐EAS demand equations for the base year. These equations represent the effects of temperature on daily EAS demand. After this, the equation set could be projected using the age‐gender–specific population change indices discussed in the last section. The projection process was basically the same as the projection for the yearly EAS demand, except that the projection target was an equation instead of a scalar. After collecting the projected age‐gender–specific temperature‐EAS demand equations, they could then be combined into a general equation to describe the overall situation of the population. In short, the projection can be summarized as a weighted linear combination of the age‐gender–specific temperature‐EAS demand equations, where the weights are the age‐gender–specific population ratio between the projection target year and the base year. Mathematically it can be written as follows:

(2) Yprojection target year=∑age ∑genderWage,genderConstantage,gender,base year+Bage,gender,base yearT+Bage,gender,base yearT2.

After obtaining the projected yearly EAS demand and age‐gender–specific temperature‐EAS demand equations from 2015 to 2051, the temporal changes in the projections over the 35 years could be visualized.

The analysis described in Sections 2.2.1 and 2.2.2 were conducted using MS Excel and IBM SPSS v30.

## 3. Results

**Figure 1:**
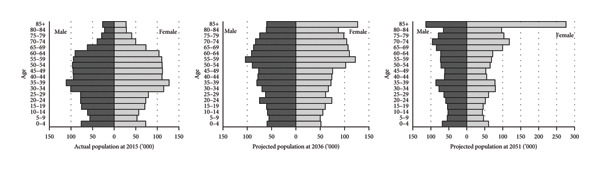
Population pyramids for Taipei city in 2015, 2036, and 2051 [30].

The official population projection for Taipei city, the actual population pyramid of 2015, and the projected population pyramids of 2036 and 2051 are presented in Figure 1. From a glance at the charts, it is immediately apparent that there is a change in the shape of the demographic structure from a rhombus in 2015 toward an inverted triangle in 2051. In addition, the left‐ and right‐hand sides of the pyramids are becoming disproportionate. The top right‐hand sides of the pyramids are becoming bigger than the left‐hand side, implying that the female population will cause a more severe problem in terms of aging than the male population. In particular, the projection suggests that the proportion of different genders in the 85+ age group in 2051 will be significantly imbalanced, with the projected female population almost 2.5 times the size of the corresponding male population.

### 3.1. Projection of Total Ambulance Demand in 2036 and 2051

Based on the official population projection data, the age‐gender–specific weighting factors for Taipei were calculated for the years 2036 and 2051, with 2015 as the base year (Table 3). From the year 2015, the projected total population only changed slightly for both genders. This can be seen from the percentage of changes in gender‐specific populations, which only ranged from −1% (male 2051) to +6% (female 2036) compared to the base year.

**Table 3:** Age‐gender–specific weighting factors for 2036 and 2051^#^.

Age group	Year 2036			Year 2051		
	Male	Female	Both	Male	Female	Both
0–64	0.86	0.81	0.84	0.76	0.66	0.71
65–74	1.66	1.72	1.69	1.77	1.78	1.78
75–84	2.61	2.69	2.66	2.77	2.91	2.85
85+	2.22	4.63	3.43	4.20	10.13	7.18

All	1.02	1.06	1.04	0.99	1.05	1.02

^#^Using 2015 as the base year.

However, when considering specific age groups, the years 2036 and 2051 registered a significant difference from the base year. For the age group below 65, the total projected population decreased by 16% and 29% for the years 2036 and 2051, respectively. Furthermore, the projected female population in this age group decreased more than the projected male population. The projected female population is expected to decrease by 19% by 2036 and 34% by 2051, whereas the corresponding figures are just 14% and 24% for the projected male population. In contrast, the age group of 65+ demonstrates a completely reversed pattern. The projected population for these categories increased remarkably, varying from a 66% increase in men aged 65–74 in 2036 to an increase of more than tenfold in women aged 85+ in 2051. Notably, the year 2051 is predicted to experience dramatic increases of 320% and 913% from 2015 in the male and female age 85+ groups, respectively. This shows that there is a possible variance based on whether we account for population structure changes when projecting EAS demand for the future.

The imbalance between the projected male and female population in each age group becomes larger and larger with the increase of age and time. In particular, for the year 2036, the projected percentage change in the male population aged 75–84 and 85+ shows a decrease of 39 percentage points from 161% to 122%, whereas the figure for the female population shows an increase of 194 percentage points from 169% to 363%. For the year 2051, the above‐projected percentage change in the population becomes even larger, with an increase of 143 and 722 percentage points for the male and female populations, respectively.

The calculation presented in Table 4 demonstrates the difference in EAS demand projection based on whether we account for age‐gender structure changes by the years 2036 and 2051. The results show that when the factor of age‐gender structure change was not considered, the projection underestimated EAS demand among people aged 65+, whereas the demand among people aged below 65 was overestimated. The level of misestimation varied between different age groups and genders. For the year 2036, the underestimation among the elderly age group ranged from 37% (men aged 65–74) to 78% (women aged 85+). In contrast, for people aged below 65, the projection registered 21% and 28% of overestimation for men and women, respectively. The overall projection of total EAS demand was underestimated by 30% if the population structure change was not considered.

**Table 4:** EAS demand in 2015 and projected demand in 2036 and 2051.

Projection year	Age group	Actual EAS demand in 2015			Projection not accounting for changes in age‐gender structure			Projection accounting for changes in age‐gender structure			Difference between the two projections		
		Male	Female	Both	Male	Female	Both	Male	Female	Both	Male (%)	Female (%)	Both (%)
2036	0–64	49,554	32,703	82,361	51,560	34,027	85,695	42,733	26,550	68,875	21	28	24
	65–74	7,366	5,441	12,815	7,664	5,661	13,334	12,225	9,359	21,691	−37	−40	−39
	75–84	8,029	7,373	15,409	8,354	7,671	16,033	20,947	19,851	40,933	−60	−61	−61
	85+	9,387	7,826	17,218	9,767	8,143	17,915	20,843	36,229	59,100	−53	−78	−70
	All	74,336	53,343	127,803	77,345	55,502	132,977	96,749	91,989	190,599	−20	−40	−30

2051	0–64	49,554	32,703	82,361	50,606	33,397	84,110	37,586	21,540	58,221	35	55	44
	65–74	7,366	5,441	12,815	7,522	5,557	13,087	13,023	9,708	22,770	−42	−43	−43
	75–84	8,029	7,373	15,409	8,199	7,530	15,736	22,260	21,482	43,959	−63	−65	−64
	85+	9,387	7,826	17,218	9,586	7,992	17,584	39,448	79,269	123,699	−76	−90	−86
	All	74,336	53,343	127,803	75,914	54,476	112,318	131,998	248,649	130,516	−32	−59	−48

For the year 2051, the results demonstrate that the pattern of misprojection would be the same as the year 2036 if the factor of age‐gender structure change was not considered and that the degree of misprojection would be more severe. The model that did not account for age‐gender structure changes caused an underestimation of 48% in the projection of total EAS demand among the whole population. The variation of the projection inaccuracy when the age‐gender structure change was accounted for ranged widely, from 35% to 90%. Specifically, the underestimation of the EAS demand for men and women aged 85+ was observed at 76% and 90%, respectively.

**Figure 2:**
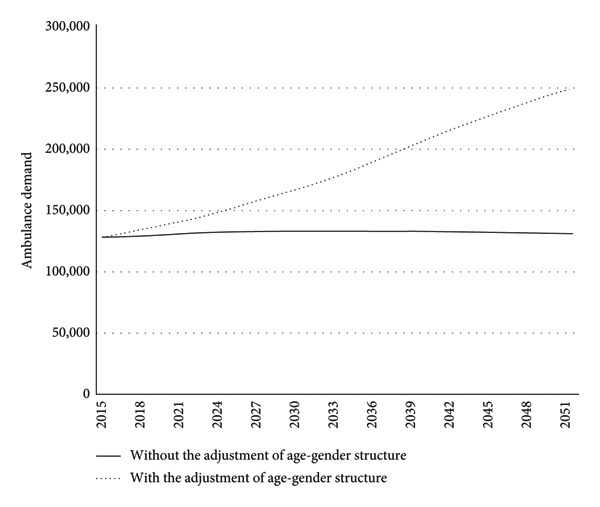
Result of the ambulance demand projections from 2015 to 2051.

To enforce the above argument, Figure 2 shows the EAS demand projections from 2015 to 2051 with and without consideration of age‐gender population structure change. It is apparent that the projected EAS demand that considered age‐gender population structure change was significantly higher than the projected EAS demand that did not consider this aspect. Moreover, the difference between the two projections was increasingly substantial over the studied period.

### 3.2. Effect of Temperature on Long‐Term Ambulance Projection Demand in Taipei

**Figure 3:**
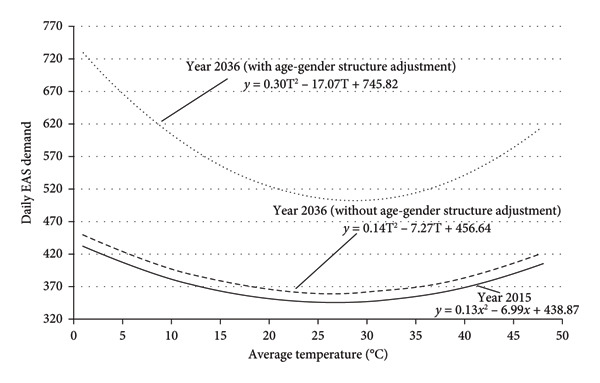
Effects of daily average temperature on EAS demand in 2036 (with and without adjustment for age‐gender structure).

**Figure 4:**
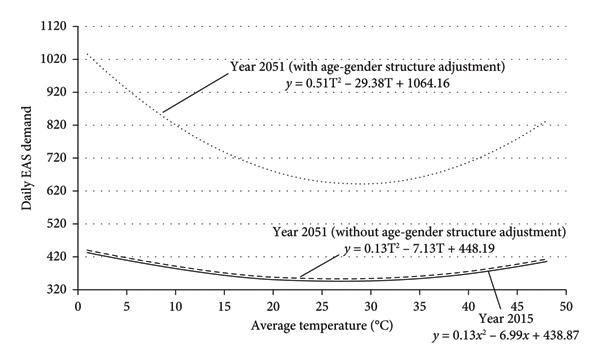
Effects of daily average temperature on EAS demand in 2051 (with and without adjustment for age‐gender structure).

The results of the regression analysis between the daily EAS demand and the average temperature in 2015 are presented in Table 5, which shows that it was the oldest age group in both genders that had the regression model with the largest adjusted *R* ^2^ (male: 0.12 and female: 0.24) compared to younger age groups. In these quartic regression models, the adjusted *R* ^2^ indicates the proportion of variance in the dependent variable (EAS demand) that can be explained by the independent variable (average temperature). The *R* ^2^ values in this analysis are not expected to be high, as the regression models were designed solely to describe the effect of average temperature, which is only one of many factors influencing EAS demand. In this study, the primary use of *R* ^2^ is for comparison between age groups. A higher adjusted *R* ^2^ reflects a stronger quartic relationship between temperature and EAS demand. Therefore, the finding that the oldest age group has a higher adjusted *R* ^2^ compared to the near‐zero values for younger groups suggests that average temperature has a greater effect on EAS demand among the oldest population. In a more vivid expression, the graphical representation of the regression models is presented in the form of a solid curve in Figures 3 and 4. The figures show daily average temperatures up to 50°C as a natural extension of the curve, simply to ensure that the figures cover a range larger than necessary. This does not imply that the author expects actual temperatures to reach 50°C.

**Table 5:** Results of age‐gender–specific regression analysis between EAS demand and daily average temperature.

Gender	Age group	Equations on 2015 data	95% CI for T	95% CI for *T* ^2^	Adj‐*R* ^2^
Male	0–64	153.31–1.88T + 0.05T^2^	(−3.82, 0.05)	(0.00, 0.09)	0.02
	65–74	25.69 − 0.37T + 0.01T^2^	(−0.94, 0.20)	(‐0.01, 0.02)	0.04
	75–84	30.24 − 0.55T + 0.01T^2^	(−1.18, 0.07)	(‐0.01, 0.02)	0.07
	85+	45.12 − 1.48T + 0.03T^2^	(−2.26, −0.71)	(0.01, 0.04)	0.12

Female	0–64	91.67 − 0.1T + 0.00T^2^	(−1.41, 1.21)	(‐0.03, 0.03)	0.00
	65–74	18.72 − 0.26T + 0.00T^2^	(−0.67, 0.16)	(‐0.01, 0.01)	0.03
	75–84	29.87 − 0.65T + 0.01T^2^	(−1.20, −0.10)	(‐0.00, 0.02)	0.12
	85+	44.25 − 1.69T + 0.03T^2^	(−2.34, −1.04)	(0.02, 0.04)	0.24

All age groups^a^		438.87 – 6.99T + 0.13T^2^			

^a^Obtained by summing the above equations.

With the year 2015 as the base year, the projected equations representing the effects of the average temperature on daily EAS demand for 2036 and 2051 are presented in Table 6. There are two equations for every age group of each gender, which represent the projections with and without consideration of age‐gender structure change. The overall projected equations for the whole population in the years 2036 and 2051 are listed as follows:

 Year 2036—with age‐gender structure adjustment: 745.82 − 17.07T + 0.30 T^2^.
 Year 2036—without age‐gender structure adjustment: 456.64 − 7.27T + 0.14 T^2^.
 Year 2051—with age‐gender structure adjustment: 1064 − 29.38T + 0.51 T^2^.
 Year 2051—without age‐gender structure adjustment: 448.19 − 7.13T + 0.13 T^2^.

**Table 6:** Results of the projected effects of daily average temperature on EAS demand for 2036 and 2051.

Gender	Age group (years)	Equations on 2036 projected data		Equations on 2051 projected data	
		With age‐gender structure adjustment	Without age‐gender structure adjustment	With age‐gender structure adjustment	Without age‐gender structure adjustment
Male	0–64	132.21 − 1.62T + 0.04T^2^	159.52 − 1.96T + 0.05T^2^	116.28 − 1.43T + 0.03T^2^	156.56 − 1.92T + 0.05T^2^
	65–74	42.64 − 0.61T + 0.01T^2^	26.73 − 0.38T + 0.01T^2^	45.43 − 0.65T + 0.01T^2^	26.24 − 0.38T + 0.01T^2^
	75–84	78.90 − 1.44T + 0.02T^2^	31.47 − 0.57T + 0.01T^2^	83.84 − 1.53T + 0.02T^2^	30.88 − 0.56T + 0.01T^2^
	85+	100.19 − 3.30T + 0.06T^2^	46.95 − 1.54T + 0.03T^2^	189.62 − 6.24T + 0.11T^2^	46.08 − 1.52T + 0.03T^2^

Female	0–64	74.42 − 0.08T + 0.00T^2^	95.38 − 0.10T + 0.00T^2^	60.38 − 0.07T + 0.00T^2^	93.61 − 0.10T + 0.00T^2^
	65–74	32.20 − 0.45T + 0.01T^2^	19.48 − 0.27T + 0.00T^2^	33.40 − 0.46T + 0.01T^2^	19.12 − 0.26T + 0.00T^2^
	75–84	80.43 − 1.75T + 0.03T^2^	31.08 − 0.68T + 0.01T^2^	87.04 − 1.90T + 0.03T^2^	30.51 − 0.66T + 0.01T^2^
	85+	204.83 − 7.82T + 0.13T^2^	46.04 − 1.76T + 0.03T^2^	448.17 − 17.11T + 0.29T^2^	45.19 − 1.72T + 0.03T^2^

All age groups^a^		745.82 – 17.07T + 0.30T^2^	456.64 − 7.27T + 0.14T^2^	1064 − 29.38T + 0.51T^2^	448.19 − 7.13T + 0.13T^2^

^a^Obtained by summing the above equations.

The above equations are visualized in Figures 3 and 4 using dashed lines in order to facilitate comparison with the solid lines, which represent the equation for the base year.

All the curves in Figures 3 and 4 show that the changes in daily average temperature are associated with variation in EAS demand. In accordance with the U‐shaped relationship, the EAS demand tends to increase with lower and higher average temperatures at both ends and stays lowest at a temperature around 25°C–30°C. The dashed curves in the figures represent the projected EAS demand in 2036 and 2051 with and without consideration of age‐gender structure. The curves of projected EAS demand without adjustment of age‐gender structure share the same pattern as the curve for EAS demand from the base year, except for a marginal upward shift. In contrast, the curves of projected EAS demand with adjustment of age‐gender structure show a significant upward shift with a larger curvature compared to the curves for the base year.

## 4. Discussion

The necessity and importance of conducting long‐term EAS demand projections are undeniable, especially as they inform health policy formulation in territories with rapidly aging populations. This study presented long‐term projections for EAS demand in Taipei city for the years 2036 and 2051, directly addressing the challenges posed by population aging. The projection methodology followed the approach developed by Lai and Wong [24], which was successfully applied in Hong Kong—a city comparable to Taipei in terms of culture and demographic structure.

By explicitly incorporating changes in age‐gender structure, this study achieved a notable improvement in the accuracy and realism of EAS demand projections compared to models that omit this critical factor. The results robustly demonstrate that demographic transitions—particularly shifts in the age and gender composition—have a substantial impact on the future trajectory of EAS demand. Consistent with Lai and Wong’s findings in Hong Kong [24], our results confirm that failing to account for demographic structure leads to significant underestimation or overestimation across population segments. However, this study also highlights a distinct local pattern, whereas Hong Kong projects a significant female‐to‐male imbalance among the middle‐aged population; Taipei’s most pronounced gender imbalance is concentrated in the 85+ age group. This demographic feature produces remarkable differences in projected EAS demand by gender, exemplified by a 76% and 90% underestimation for men and women, respectively, in 2051. Such findings underscore the necessity of gender‐sensitive and age‐specific planning for EAS and allied health resources, to adequately meet the growing demand for healthcare among older adults.

In addition to demographic factors, this study’s investigation of temperature effects provides important insights for both theory and policy. The projection results confirmed that extreme temperatures—both low and high—will have an increasingly severe impact on EAS demand in future decades, with the effect amplified by the aging of the population. The U‐shaped relationship identified between daily average temperature and EAS demand illustrates that as the population ages, the system will become even more sensitive to temperature extremes. These results carry direct policy implications: health service planners and emergency systems must prepare for amplified seasonal and weather‐related surges in demand, particularly in periods of heatwaves or cold spells. Moreover, the analysis revealed that, among those under 65, the regression models yielded low adjusted *R* ^2^ values, suggesting that factors beyond temperature—potentially including acute illnesses, accidents, or behavioral trends—may play a larger role in shaping demand in these groups. This highlights a potential avenue for further research.

The observed underestimation of the curvature of the regression line for daily average temperature and EAS demand, even when only temperature is considered, provides additional evidence for the crucial influence of demographic change. Policymakers must recognize that ignoring the aging population issue, especially during periods of extreme weather, would present significant risks to the adequacy and responsiveness of EAS systems.

Looking ahead, enhancing projection models by incorporating additional factors strongly associated with EAS demand is a promising strategy for future research and practice. Leveraging advanced analytical methods and algorithms may further increase projection accuracy and support more precise resource planning. The study by Sasaki et al. [25], for example, demonstrated how genetic algorithms—applied to long‐term EAS demand projections—can be used to optimize ambulance locations, thereby improving emergency response times and patient outcomes. Similarly, the research by Veser et al. [7]on demographic impacts in Bavaria, Germany, illustrates how recognizing regional variation in EAS demand can inform more effective, equitable resource allocation across urban and rural areas.

While emerging artificial intelligence techniques, such as deep learning, offer considerable potential for improving the predictive accuracy of long‐term projections, the strength of the current approach lies in its methodological simplicity and transparency. These attributes are particularly critical for governmental agencies—such as the Taipei City Fire Department—that require projection tools which are both stable and interpretable. In contrast, deep learning, often described as a “black‐box” approach, may be less appropriate in settings where decision makers must clearly justify and communicate methods to the public and other stakeholders [31]. Furthermore, the objective of long‐term projections is to identify and monitor directional trends, rather than to achieve perfect point estimates—a focus fundamentally different from that of short‐term forecasting, which prioritizes precision at the individual level. Therefore, a modest reduction in predictive accuracy may be acceptable if projections are periodically updated as new data become available.

This study makes several significant contributions to the literature on emergency medical service planning and health policy in aging societies. First, it is among the first to present an integrated long‐term projection framework for EAS demand in an Asian metropolis that simultaneously accounts for demographic transitions and climate‐related factors. By explicitly modeling the impact of age‐gender structural change and temperature extremes, the study provides a more realistic and policy‐relevant estimate of future ambulance service needs. Second, the application of a transparent and well‐documented projection method previously applied in Hong Kong enables meaningful cross‐regional comparison and benchmarking, strengthening the external validity and transferability of the findings. Third, the results offer timely empirical evidence for Taipei city, a setting facing both rapid aging and increasing climate variability, thus filling an important gap in the Asian urban health systems literature. These projections offer actionable guidance for governmental agencies in strategic workforce planning, resource allocation, and emergency preparedness. Fourth, the study demonstrates the practical utility of maintaining a balance between methodological rigor and transparency. While emerging AI approaches hold promise for future refinement, this research illustrates the current advantages of using interpretable models that can be clearly communicated to stakeholders and policymakers. Finally, by highlighting the compounding effects of demographic and environmental change, the study sets a foundation for future research to further refine demand forecasting models, incorporate additional relevant variables, and extend the analysis to other urban and regional contexts. Taken together, these contributions support the development of resilient, equitable, and sustainable emergency healthcare systems in aging and climate‐affected societies.

## 5. Limitations

The first limitation of this study concerns the limited generalizability of the projection results. Specifically, the findings are based on data from Taipei city—the most urbanized region in Taiwan—and may not be directly applicable to rural or less densely populated areas, where demographic changes may follow different patterns. While the projection methodology itself is transferable to other regions, the specific numerical results and implications should be extrapolated with caution.

Secondly, this study focused solely on demographic structure change, assuming that other systemic factors—such as the legal framework, medical technology, and disease evolution—remain constant over time. We acknowledge that these assumptions may not hold in the long term; however, due to the unpredictability and complexity of modeling such factors, they were not included in the current framework. In addition, some confounding factors, such as education level and socioeconomic status, could not be incorporated because only essential information was collected by paramedics. The aim of this research was to equip policymakers with a foundational model for understanding the long‐term trends in EAS demand driven by an aging society and climate change. We have established this baseline. The clear next step is for future studies to build upon this framework. By integrating a wider array of variables, researchers can enhance the model’s predictive power and provide more nuanced insights. Such an expanded model would be invaluable for guiding critical operational planning, including the optimization of EAS depot locations and the strategic scheduling of manpower to meet future needs.

Finally, the difficulty in validating the projection results also represents a limitation of this research. Since the effects of population structure change will only become apparent after more than a decade, data to validate the model are not currently available. As previously mentioned, the main aim of the projection is to emphasize the identification of directional trends rather than precise point estimates. Most previous research in this area has also faced similar challenges in addressing this limitation [7, 24, 25].

## 6. Conclusion

In this study, over 140,000 EAS patient records from 2015 were used to project future EAS demand, taking into account changes in the age‐gender structure relative to the base year. Furthermore, the relationship between daily average temperature and EAS demand in 2036 and 2051 was examined, both with and without incorporating age‐gender structure changes. The results revealed that failing to consider age‐gender structure leads to substantial underestimation of demand among older adults and overestimation among younger groups, as well as notable shifts in the temperature–demand relationship. These findings highlight the critical importance of accounting for demographic change in long‐term EAS demand projections.

With accurate long‐term projections for EAS demand in Taipei city and other regions in Taiwan, the government can develop effective strategies to improve the EAS system in response to the rapidly aging population. Projections should be detailed and should not exclude key factors that significantly influence EAS demand; otherwise, inadequate preparation could have serious consequences, as EAS is a service directly related to life and death.

## Ethics Statement

This study only analyzed anonymous EAS usage records, with no direct or indirect patient contact. The project has been certified for exemption from the Human Research Ethics Committee at National Cheng Kung University (No. 108–298).

## Conflicts of Interest

The authors declare no conflicts of interest.

## Author Contributions

H.T.W. and T‐D.N. contributed equally to this study.

## Funding

This work was supported by the National Taiwan Normal University and a grant from the National Science and Technology Council, Taiwan (NSTC 112–2410‐H‐003–083‐).

## Data Availability

The data that support the findings of this study are available from the authors with the permission of the Taipei City Fire Department and the Central Weather Bureau. Restrictions apply to the availability of these data, which were used under license for this study.
